# Assessment of Ultraviolet-C Light for Sterilization of Hysteroscopy Instruments

**DOI:** 10.7759/cureus.73609

**Published:** 2024-11-13

**Authors:** Jessica A Mora-Galván, Luis F Escobar-Ponce, Andrea Olguín-Ortega, Graciela Villeda-Gabriel, Ricardo Figueroa-Damián, Alejandro Rendón-Molina

**Affiliations:** 1 Department of Gynecology, National Institute of Perinatology, Mexico City, MEX; 2 Department of Infectology and Immunology, National Institute of Perinatology, Mexico City, MEX; 3 Research Division, National Institute of Perinatology, Mexico City, MEX

**Keywords:** hysteroscopy, infection control, medical instruments, sterilization, ultraviolet c

## Abstract

Objective

To evaluate the sterilization efficacy of hysteroscopy instruments using ultraviolet C (UV-C) light at a wavelength of 259 nm in the Endoscopic Diagnostic Center of the National Institute of Perinatology.

Methods

Consecutive patients undergoing office hysteroscopy via the Bettochi vaginoscopy technique were included, excluding those with conditions such as viable intrauterine pregnancy or acute pelvic infection. Samples were collected from six designated sites of the hysteroscope, including the inner sheath, internal holes of the inner sheath, lens, graspers, scissors, and outer sheath. Initially, samples were taken after the first sterilization using a LAOKEN LK/MJG-150 Plasma Sterilizer (Chengdu, China). Next, samples were collected after the routine use of the hysteroscope in the office setting to confirm contamination. Subsequently, a new set of samples were taken after a 20-minute UV-C sterilization cycle with the EsteriUV device.

Results

The initial sterilization achieved a 96.73% sterilization rate, with ten samples testing positive for *Staphylococcus* *coagulase*-negative. Post-hysteroscopy, contamination increased significantly. Afterward, UV-C sterilization achieved a 96.08% sterilization rate, with 11 samples positive for *Staphylococcus* *coagulase*-negative and one for *Streptococcus anginosus* (p=0.66). No clinical infections were reported in any patient within one-month post-procedure.

Conclusion

UV-C light is a viable alternative for hysteroscopy instrument sterilization, demonstrating comparable efficacy to conventional methods. Further studies are recommended to optimize UV-C parameters for enhanced sterilization efficiency.

## Introduction

Hysteroscopy involves inserting a rigid or flexible hysteroscope into the uterus and using distending media for clear visualization, enabling minimally invasive diagnosis and treatment of endocervical and intrauterine issues [1]. Hysteroscopy employing a vaginoscopic approach remains the preferred and gold standard technique, noted for its safety and patient comfort [2]. Infection rates after hysteroscopy were minimal with and without antibiotics (0.52% vs. 0.58%), with rare serious infections (PID or abscess) in pretreated patients (0.2%) and none in controls [3]. Infections can arise from procedural complications due to microorganisms from the patient (endogenous) or from contaminated instruments (exogenous source) [4]. Preventing surgical site infections (SSIs) reduces patient mortality and alleviates the strain on the national healthcare system [5].

Despite global, national, and regional guidelines for endoscope reprocessing, contamination, and microorganism transmission persist, primarily attributed to equipment defects, reprocessing failures, and failure to adhere to prescribed protocols [6]. Facilities performing endoscopy should prioritize minimizing pathogen transmission by enabling the implementation of surveillance strategies to enhance reprocessing quality [7].

Healthcare facilities achieve sterilization through various physical or chemical methods, using agents like steam, dry heat, ethylene oxide gas, hydrogen peroxide gas plasma, vaporized hydrogen peroxide, and liquid chemicals [8]. Initially, sterilizing and cleaning reusable items were thought to be more environmentally responsible than single-use items. However, a review showed that single-use plastics have a greater environmental impact over their lifespan [9]. On a national level, manufacturers can be influenced to prioritize environmentally friendly, sterilizable, and reusable instruments over single-use plastics, with a focus on recycling and refurbishment, while also advocating for the removal of endocrine-disrupting chemicals (EDCs) from all medical devices [10]. The UV light system offers the most economical solution for reprocessing medical devices in large quantities, as described by Biadsee in 2023 [11]. Currently, there is considerable interest in utilizing ultraviolet light-emitting diodes (UV LEDs) and excimer lamps, which emit within the UV-C spectral range (180-280 nm), due to advancements in lamp technologies [12].

The primary factors governing UV-C disinfection include wavelength, dosage, relative humidity, and temperature, with no universally agreed-upon optimal values; however, in most cases, effective disinfection entails high-dose exposure to a spectrum of wavelengths, notably around 260 nm, within a room temperature environment characterized by low relative humidity [13]. UV radiation (UVR) is known for its virucidal properties, damaging viral genomes by inducing pyrimidine dimers and generating reactive oxygen species (ROS), which inactivate microorganisms and inhibit replication [14]. One of the most recent studies published in 2024 has demonstrated that UV-C light disinfection for flexible endoscopes without a working channel appears to be more effective at reducing CFUs than the Endoscope Washer Disinfector, suggesting it may serve as an effective alternative disinfection method [15].

This study aimed to assess the sterilization efficacy of hysteroscopic instruments used during procedures in the Endoscopic Diagnostic Center of the National Institute of Perinatology using ultraviolet C spectrum rays at 259 nm (ESTERIUV ultraviolet light equipment [Paraísos del Colli, Mexico]).

## Materials and methods

Participants

Inclusion criteria comprised consecutive patients undergoing office hysteroscopy procedures using the vaginoscopy technique (Bettocchi [Tuttlingen, Germany]) at the Diagnostic Endoscopy Center of the National Institute of Perinatology, as per their clinical requirements determined by the Gynecology and Reproduction services. Exclusion criteria encompassed patients with viable intrauterine pregnancy, acute pelvic infection (including pelvic inflammatory disease, active or prodromal herpes), known cervical or uterine cancer, recent uterine perforation, or excessive uterine bleeding. Additionally, elimination criteria were applied to patients lacking microbiological cultures of the hysteroscopy instruments used or failing to attend a clinical evaluation one month post-hysteroscopic procedure. No participants with active endometritis were included.

Outcomes

Sterilization is the process of eradicating all viable microorganisms and their germinative elements, such as spores, endospores, and eggs [16].

Infection rate: Number of infections occurring after the hysteroscopy procedure within the evaluation month. The rate of post-hysteroscopic procedure infections using the vaginoscopic technique was investigated as an outcome parameter in the study, as well as the type of microbiological isolation. Endometritis was defined as an inflammatory condition of the endometrial lining of the uterus, characterized clinically by symptoms such as fever above 38°C, pelvic pain, and a negative urinary culture; early-onset endometritis appears within the first 48 hours following an inciting event [17].

Procedure

At the National Institute of Perinatology, hysteroscopy using the Bettocchi vaginoscopy technique was performed according to criteria by gynecology and reproduction services. Patients with viable intrauterine pregnancy, acute pelvic infection, active herpes, known cervical or uterine cancers, recent uterine perforation, or heavy bleeding were excluded. Instruments were sampled for microbial cultures before and after sterilization with the LAOKEN LK/MJG-150 Plasma Sterilizer, and six sites were sampled from the hysteroscope. After the procedure, tools were cleaned with surgical soap, air-dried, and sterilized with the 'EsteriUV' device for 20 minutes. Samples were collected again for microbial analysis. Patients were prescribed azithromycin and ketorolac before the procedure. The medical team introduced themselves, explained the procedure, and obtained informed consent. Patients were gowned and asked to empty their bladders. The hysteroscopy equipment was checked and assembled, including a 2.9 mm optic hysteroscope, continuous flow sheath, scissors, biopsy forceps, bipolar electrodes, and more. The visualization system included cold-light xenon optics, an HD camera head, fiber optics, a recording system, and a medical-grade monitor. The patient was positioned, and the procedure began with a white balance adjustment. Saline solution was used for distension, ensuring intrauterine pressure did not exceed 100 mmHg and saline volume stayed below 1500 cc. The hysteroscope was inserted using an atraumatic vaginoscopic approach, exploring and visualizing the uterine structure, avoiding wall contact, and ensuring continuous fluid flow. Cervical stenosis was managed with a grasper or cold cut with scissors. Diagnostic hysteroscopy lasted 5 to 15 minutes; if exceeded, the procedure was paused and rescheduled. The session included biopsy, polypectomy, myomectomy, or resection of synechiae, uterine septum, or removal of foreign bodies. The procedure concluded with the careful withdrawal of the hysteroscope and UV light sterilization.

UV sterilization

The cleaning and sterilization process of the equipment begins by rinsing the instruments under a direct stream of water for 15 seconds to remove organic residues. Subsequently, the instruments are scrubbed in an up-and-down motion (from entry to exit) using a brush and surgical soap for 60 seconds. Internal cleaning of the instruments involves using a thin brush to scrub inside the working channels; the brush is rotated three times in a clockwise direction, and surgical soap is used. After scrubbing, the instruments are rinsed again under a direct stream of water, allowing the water to flow from top to bottom (entry to exit) for another 15 seconds. The instruments are then dried from top to bottom (entry to exit) and further dried using compressed air from the main entry point to the exit. Once dried, the instruments are placed on a tray for sterilization. The tray was then introduced into the sterilization unit (EsteriUV) and set for a 20-minute cycle. After the cycle, the tray with the instruments is removed using sterile technique and sterile gloves.

Microbiological procedure

For the culture of the different evaluated surfaces of the hysteroscope, the following technique was followed:

A sterile swab with a cotton tip moistened with sterile saline solution was used to sample the surface of each hysteroscope element to be cultured.

After collecting the sample, the swab was placed into a tube containing Brain Heart Infusion (BHI) broth and transported to the microbiology laboratory. The tubes were then incubated at 37°C for 72 hours, and the possibility of bacterial growth was checked every 24 hours. Broths showing turbidity were plated on the following culture media: sheep blood agar, salt and mannitol agar, MacConkey agar, and potato dextrose agar.

Inoculated agar plates were incubated at 37°C for 48 to 72 hours. Sheep blood agar plates were incubated in a 5% CO2 incubator at 37°C for 72 hours. Plates showing growth on culture media were examined for colony morphology, subjected to Gram staining, and tested for oxidase and catalase activity. Identification tests were performed by selecting cards based on microscopic morphology using the Vitek automated system, which provides a report that includes microorganism identification and an antimicrobial susceptibility profile upon completion of the procedure.

Sample size

We calculated the required sample size based on expected effectiveness rates of 97% for the plasma sterilizer and 99.7% for the UV method. We employed a sample size calculation formula for comparing two proportions in a superiority framework to detect a statistically significant difference between the two methods. Using this formula, we determined that 275 samples per group are necessary to achieve a study power of 80% with a significance level of 0.05 for a total of 550 participants to adequately test the hypothesis that the UV sterilization method is superior [18]. This calculation considers the desired power to detect a 0.7% increase in effectiveness, which is clinically significant for our study. We included 306 samples in each method of sterilization.

Ethical considerations

Since this study did not involve any procedure in patients, it was considered a risk-free study; for this reason, it was not subjected to the Institutional Review Board. However, in all cases, informed consent was collected from the patients undergoing hysteroscopy to take samples for microbial cultures of the different components of the hysteroscope used.

## Results

In our assessment of the sterilization efficacy, 10 out of 306 samples (approximately 3.27%) tested positive for *Staphylococcus coagulase*-negative from the initial sterilization process, as shown in Table 1. Of these positive samples, five were from the inner sheath, one from the internal orifice of the inner sheath, two from the lens, two from the grasper forceps, and one from the outer sheath. Following the hysteroscopic procedures, subsequent cultures were retaken from identical sites, which revealed a comprehensive microbial profile. Following the second sterilization procedure utilizing UV light, the microbial assessment revealed 11 positive cultures for *Staphylococcus coagulase*-negative and one for *Streptococcus anginosus*. Out of the total samples processed, these results yield a sterilization rate nearly identical to that achieved by the initial method.

**Table 1:** Sterilization rate *All isolated bacteria following the hysteroscopy procedure are shown in Table 2. **Chi square test

Stage description	Total samples	Positive for Staphylococcus coagulase-negative	Positive for Streptococcus anginosus	Positive for other bacterias*	Sterilization rate	p**
First Sterilization Procedure	306	10	0	0	96.73%	
After Hysteroscopic Procedure	306	124	4	152	-	
After UV Sterilization Method	306	11	1	0	96.08%	0.66*

Our analysis involved 306 samples for each procedure to evaluate sterilization effectiveness across different methodologies. The initial sterilization procedure achieved a sterilization rate of 96.73%, with only ten samples testing positive for *Staphylococcus coagulase*-negative. Following the hysteroscopic procedure, there was a notable increase in bacterial presence, with 124 samples positive for *Staphylococcus coagulase*-negative, 4 for *Streptococcus anginosus*, and 33 for other bacteria, indicating the contamination of the hysteroscope after the procedure. Subsequent sterilization using the UV light method closely matched the efficacy of the initial procedure, achieving a sterilization rate of 96.08%. The comparison between the first sterilization and the UV method, evaluated using a Chi-square test, yielded a p-value of 0.66, indicating no statistically significant difference in effectiveness, these results were comparable across the different sterilization methods. These results suggest that both sterilization methods are comparably effective, with the UV light method offering a viable alternative with similar outcomes.

Table 2 describes the bacteria found after the hysteroscopy. The most frequently encountered bacterium was *Staphylococcus coagulase*-negative, with a total count of 124 instances across all device parts, followed closely by Enterococcus faecalis, which was detected 88 times. Escherichia coli was found 25 times, indicating a notable presence, albeit significantly lower than the top two bacteria. Additionally, bacterial combinations were noted.

**Table 2:** Sampled bacterium after the use of the hysteroscope

Bacteria	INNER SHEATH	INTERNAL HOLES OF INNER SHEATH	LENS	GRASPERS	SCISSORS	OUTER SHEATH	TOTAL
*Staphylococcus coagulase* negative	21	22	20	21	19	21	124
Enterococcus faecalis	16	18	13	14	14	13	88
Escherichia coli	6	5	5	4	1	4	25
Lactobacillus spp.	0	2	2	0	0	0	4
Klebsiella pneumoniae	0	0	1	0	0	0	1
Streptococcus anginosus	1	1	1	1	0	0	4
Gram-negative bacteria	1	0	0	0	0	0	1
Gram-positive bacteria	1	0	0	0	0	0	1
*Enterococcus faecalis *and *Staphylococcus coagulase* negative	6	6	4	5	1	4	26
*Escherichia coli *and *Enterococcus faecalis*	1	1	0	0	0	0	2
*Escherichia coli* and *Staphylococcus coagulase* negative	1	1	0	0	0	0	2
*Staphylococcus coagulase* negative and *Lactobacillus spp.*	0	1	0	0	0	0	1
*Staphylococcus coagulase* negative and *Streptococcus anginosus*	0	1	0	0	0	0	1

All patients underwent clinical evaluation one month following their hysteroscopy procedure. None of the patients exhibited signs of clinical vaginal or uterine infection post-procedure. Each patient was instructed on the symptoms of a potential infection and advised to seek immediate assessment at the emergency services should they suspect an infection. However, there were no reported infections in either group.

## Discussion

UV light has been extensively studied for its disinfection capabilities against bacteria, viruses, and spores. Specifically, UV-C light has proven effective as a valuable complement to terminal manual cleaning protocols in hospitals due to its effectiveness as a germicidal agent, particularly in high-traffic and high-touch areas with significant bioburden [19]. Recent research has demonstrated that a UV disinfection process, when applied for 35 seconds, exhibits a significantly higher sporicidal efficacy compared to FDA-approved chemical sterilizants [20]. Like our tested equipment, the ZAPARAY™ UVC LED chamber has proven to be a time- and energy-efficient disinfection alternative, achieving a 9 log10 bacterial reduction on Petri dishes and varying degrees of reduction on contaminated medical devices. This indicates its potential as a standardized substitute for specific manual disinfection methods, provided that efficacy testing is conducted for each type of instrument [21]. Uncontrolled antibiotic usage has spurred the proliferation of antibiotic-resistant bacteria, prompting research into UV light-emitting diodes as effective tools for deactivating such microorganisms, with optimal inactivation observed at 265 nm wavelength [22].

The results of our study demonstrate that the UV light sterilization method achieved a substantial sterilization rate of 96.08%, which is closely aligned with the efficacy observed in the initial sterilization procedure. These findings are significant, as previous studies reported lower inactivation rates for various pathogens under different UV-C conditions. For example, a study found 90% inactivation for *E. coli*, *Pseudomonas fluorescens*, and *Listeria innocua* at UV-C doses of 1.5 to 1.9 mJ/cm² across wavelengths. Our higher sterilization rate suggests that our UV-C dosage and wavelength parameters are more effective for pathogens in hysteroscopic instruments [23].

Another aspect to consider is that biofilm formation on reusable medical device surfaces is a risk that can be controlled. By ensuring prompt device cleaning and reprocessing, either by high-level disinfection or sterilization and proper drying, biofilms will not have a chance to form [24]. As per the positive cultures, biofilm formation greatly enhances bacterial survival on hospital surfaces, conferring high resistance to desiccation, benzalkonium chloride disinfection, and UV radiation [25]. The *Staphylococcus coagulase*-negative bacterium forms a biofilm; its threat lies in its ability to produce extracellular matrix polymeric substances and other mechanisms that may enhance resistance to UV light [26]. Biofilm communities, evolving over billions of years, protect microbial cells by limiting UV radiation penetration to the top layers, where cells can produce compounds like mycosporine-like amino acids and carotenoid pigments for additional defense [27].

The effects of various UV wavelengths and their combinations on the inactivation of microorganisms have been studied, finding that while 265 nm UVC LED exhibits greater DNA-damaging potential, 280 nm radiation effectively represses photoreactivation and dark repair mechanisms [28]. The consistent sterilization outcomes, despite varying microorganism sensitivity to UV, show the robustness of our UV sterilization. Another study reduced endoscope contamination in otorhinolaryngology from 66.908 (± 239.215) CFU to 0.12 (± 0.39) CFU, with 10% minimally contaminated, mainly with normal skin flora, similar to our *Staphylococcus coagulase*-negative results [29].

In the study of Ezeh, they aimed to compare the effectiveness of UV-C light at approximately 254 nm versus standard Cidex orthophthalaldehyde (OPA) disinfection for flexible fiberoptic laryngoscopes, finding comparable efficacy in reducing bacterial contamination [30]. In addition to various parameters affecting disinfection efficiency, challenges like shadowing and photoreactivation complicate the establishment of a universal solution, as higher UV doses improve inactivation but may also cause material damage and require longer exposure times, leaving optimal values undetermined [13].

## Conclusions

Our UV light method demonstrates high inactivation rates across a range of contaminants, reaffirming its efficacy and positioning it as a reliable alternative to conventional sterilization techniques, which is particularly crucial in procedures requiring stringent aseptic conditions, such as hysteroscopies, where any compromise in sterilization can result in significant complications.

In conclusion, our findings support the broader implementation of UV light sterilization in clinical settings, specifically tailored to address the pathogens and procedural requirements encountered. Further research is recommended to identify optimal UV-C doses and wavelengths that maximize sterilization efficacy while minimizing exposure time, thereby enhancing the safety and efficiency of medical procedures.
